# Structural basis for specific single-stranded RNA recognition by designer pentatricopeptide repeat proteins

**DOI:** 10.1038/ncomms11285

**Published:** 2016-04-18

**Authors:** Cuicui Shen, Delin Zhang, Zeyuan Guan, Yexing Liu, Zhao Yang, Yan Yang, Xiang Wang, Qiang Wang, QunXia Zhang, Shilong Fan, Tingting Zou, Ping Yin

**Affiliations:** 1 National Key Laboratory of Crop Genetic Improvement and National Centre of Plant Gene Research, Huazhong Agricultural University, Wuhan 430070, China; 2 Center for Structural Biology, School of Life Science, Tsinghua University, Beijing 100084, China

## Abstract

As a large family of RNA-binding proteins, pentatricopeptide repeat (PPR) proteins mediate multiple aspects of RNA metabolism in eukaryotes. Binding to their target single-stranded RNAs (ssRNAs) in a modular and base-specific fashion, PPR proteins can serve as designable modules for gene manipulation. However, the structural basis for nucleotide-specific recognition by designer PPR (dPPR) proteins remains to be elucidated. Here, we report four crystal structures of dPPR proteins in complex with their respective ssRNA targets. The dPPR repeats are assembled into a right-handed superhelical spiral shell that embraces the ssRNA. Interactions between different PPR codes and RNA bases are observed at the atomic level, revealing the molecular basis for the modular and specific recognition patterns of the RNA bases U, C, A and G. These structures not only provide insights into the functional study of PPR proteins but also open a path towards the potential design of synthetic sequence-specific RNA-binding proteins.

## Abstract (web summary)

Pentatricopeptide repeat (PPR) proteins bind RNA and are involved in the regulation of RNA metabolism in eukaryotes. Here, the authors examine the capability of these proteins as modules for gene manipulation using structural biology methods.

## Graphical abstract

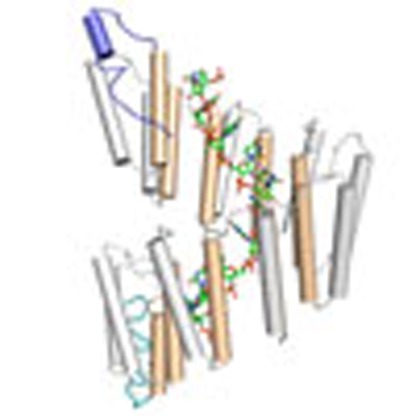

The ability to design proteins to manipulate specified target DNA/RNA sequences has been a long-sought but elusive goal^1^. The modular mode of target recognition by specific proteins allows the development of DNA/RNA-binding tools through the assembly of particular motifs or domains^2^. Despite encouraging progress in the realm of DNA editing^1^, including, for example, the successful application of zinc finger domains^3^, transcription activator-like effectors (TALE)^4^,^5^ and the clustered regularly interspaced short palindromic repeat (CRISPR)–Cas9 system^6^,^7^ in targeted gene regulation, our knowledge of how to design proteins that can selectively bind desired RNA sequences remains limited. Pumilio and FBF homology (PUF) family proteins contain an RNA-binding domain that characteristically comprises eight α-helical repeats, each of which recognizes one RNA base^8^. The application of the PUF domain involves eight/sixteen repeats, each binding a specific nucleotide, to generate RNA-recognition tools^9^,^10^. Nevertheless, there are limits to the further application of engineered PUF domains^2^. Thus, more candidates with a potential for RNA manipulation are required to enrich the gene-regulation toolbox. Containing a tandem array of 2–30 repeats, pentatricopeptide repeat (PPR) proteins constitute one of the largest protein families in land plants^11^,^12^. Each PPR repeat aligns to a single nucleotide of the RNA target in modular patterns, making these proteins suitable for the development of exciting new biotechnologies^13^,^14^,^15^,^16^.

A typical PPR repeat, also referred to as a PPR motif^17^, is defined as a degenerate 35-amino-acid repeat (hence the term pentatricopeptide) that folds into a hairpin of antiparallel alpha helices, as revealed by the crystal structures of the PPR domains of protein-only RNase P1(ref. 18) from *Arabidopsis thaliana* and of human mitochondrial RNA polymerase^19^,^20^, and by the structures of the RNA-bound PPR repeat assembly of PPR10^21^,^22^ and Thylakoid assembly 8 (THA8)^23^,^24^. Within each repeat, the combinations of two amino acids, known as the PPR code at two key positions (the 5th and the 35th) confer RNA specificity^15^,^25^,^26^. The structure of the *PSAJ–*PPR10 complex corroborates this binary code model^22^, demonstrating that these two amino acids interact directly with the target nucleobases. More than 30 combinations of PPR code amino acids, such as ‘ND (asparagine and aspartate)' and ‘TD (threonine and aspartate)', have been predicted by bioinformatic studies^25^,^26^,^27^. However, the specific nucleotide targets binding to them have been only partially identified by biochemical and structural studies^21^,^22^,^28^,^29^,^30^. Until now, the PPR code remains largely enigmatic, requiring further elucidation^15^,^26^.

In the past decade, many PPR genes have been identified and extensively studied^15^,^26^. PPR proteins function in various aspects of RNA metabolism, primarily in organelles, facilitating the editing^31^, processing^32^, splicing^33^ and translation of RNAs^34^. However, most of their functions remain unclear because their target RNA sequences are incompletely understood. Deciphering the PPR code will greatly facilitate precise RNA target prediction and identification and the functional investigation of PPR proteins, which requires elaborate structural information about the PPR–RNA complex^15^.

Previously, our group has successfully designed 35-amino-acid PPR repeat scaffolds, determined via comprehensive computational homology analysis of P-type PPR proteins from *A. thaliana*^28^. We assembled PPR repeat scaffolds in tandem and fused parts of PPR10 from *Zea mays* onto the amino and carboxyl termini. Engineered designer (dPPR) proteins can specifically select their predicted RNA targets according to PPR codes *in vitro*. Artificial manipulation of the PPR code amino acids, rather than the PPR repeat scaffolds, leads to specific RNA recognition. Similar results and crystal structures of artificially engineered PPR proteins free of target RNA have also been reported by other groups^29^,^35^. These results suggest that PPR scaffolds are amenable to the engineering of designer RNA-binding domains, as promising tools to achieve specific RNA recognition *in vitro*. Despite these advances, the lack of a structure of a dPPR–RNA complex has hindered the elucidation of RNA recognition by dPPR proteins and, more importantly, has restricted the development of more efficient RNA manipulation tools.

To determine how dPPR proteins specifically accommodate and recognize their single-stranded RNA (ssRNA) targets, we designed and purified a set of homogeneous dPPR proteins with high-target recognition specificity and solved the crystal structures of four different dPPR proteins in complex with their respective target ssRNAs. A typical RNA-bound dPPR protein adopts a right-handed superhelical spiral structure, with its target ssRNA molecule sitting in the interior cavity and interacting with corresponding PPR repeats in a modular fashion. These structures also reveal atomic-level interaction patterns among all four RNA bases (U, C, A and G) with different PPR codes. On the basis of our structural discoveries, models of RNA recognition by some additional PPR codes were verified. Together, our findings not only provide detailed modular and specific binding patterns of dPPR repeat, but also establish an important framework for gene-manipulation applications.

## Results

### RNA sequence selectivity of dPPR proteins

**Figure 1:**
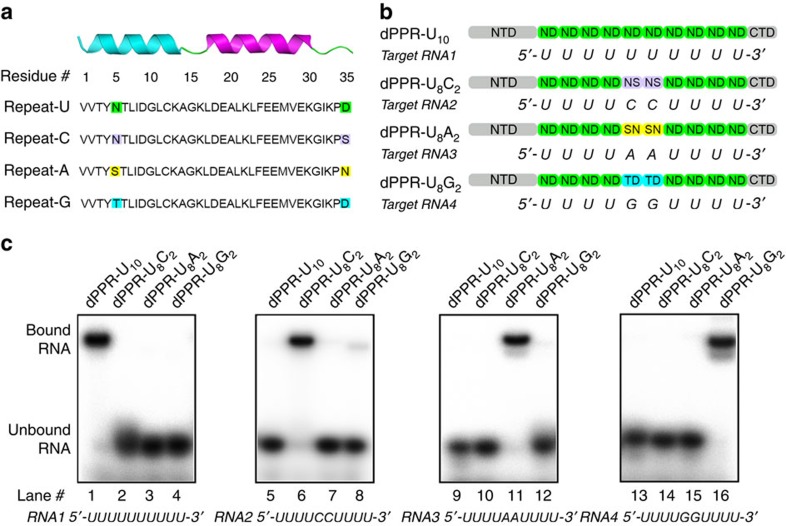
Design of dPPR proteins with RNA-recognition specificity. (**a**) Sequence of dPPR motif containing 35 amino acids. The secondary structural elements of a typical PPR motif are shown above. PPR codes comprising two residues located at the 5th and 35th positions are labelled in distinct colours (‘ND', ‘NS', ‘SN' and ‘TD', which recognize uracil, cytosine, adenine and guanine, respectively, are coloured green, lilac, yellow and cyan, respectively). Single-letter abbreviations for the amino acid residues are as follows: A, Ala; C, Cys; D, Asp; E, Glu; F, Phe; G, Gly; I, Ile; K, Lys; L, Leu; M, Met; N, Asn; P, Pro; S, Ser; T, Thr; V, Val and Y, Tyr. (**b**) Schematic representation of dPPR-U_10_, dPPR-U_8_C_2_, dPPR-U_8_A_2_ and dPPR-U_8_G_2_, and their targeting of specific RNA sequences. The shaded binary amino acids indicate PPR repeats with different codes. The NTD and CTD are from native PPR10. (**c**) Specific RNA target binding of dPPRs. In the RNA EMSA, 50 nM purified dPPRs were mixed with 2 nM ^32^P-labelled RNA, respectively. The sequence of the RNA probe is listed below each panel. Fig. 1a is reprinted from, Shen *et al.*^28^ with permission from Elsevier.

On the basis of previous investigation^28^, we used dPPR scaffolds with four different codes: ND, NS, SN and TD (Fig. 1a). Each of the dPPR repeat scaffolds contains 35 amino acids, and those at positions 5 and 35 are referred to as PPR code amino acids. Parts of PPR10 from *Z. mays* were fused onto the amino and carboxyl termini of a series of tandem dPPR repeats as amino-terminal domain (NTD) and carboxyl-terminal domain (CTD), to enhance the solubility of the engineered protein (Fig. 1b and Supplementary Fig. 1). To verify the RNA selectivity, the dPPR proteins dPPR-U_8_N_2_ (in which N indicates any nucleotide) comprising 10 dPPR repeats with different 5th and 6th repeats with different PPR codes were constructed and purified to homogeneity. Each of the four dPPR-U_8_N_2_ proteins specifically bound to its respective target ssRNA with a dissociation constant of ∼20 –75 nM, as estimated on the basis of the results of electrophoretic mobility shift assay (EMSA) (Fig. 1c; Supplementary Fig. 2 and Supplementary Table 1). The substitution of any target RNA base with another led to a notable reduction in or complete abrogation of dPPR-U_8_N_2_ binding (Fig. 1c and Supplementary Fig. 2). For instance, the substitution of cytosine at positions 5 and 6 with the pyrimidine uracil or the purines adenine and guanine resulted in the dissociation of the dPPR–RNA complex.

Furthermore, we were able to target a specific ssRNA sequence by assembling dPPR proteins with a combination of four types of PPR codes. For example, dPPR-4, which comprises eight dPPR repeats with all four types of dPPR code, bound to its predicted target RNA with high specificity as expected. The dissociation constant was ∼25 nM (Supplementary Fig. 3 and Supplementary Table 1). In summary, dPPR proteins can distinguish target sequences with high specificity.

### Crystallization of RNA-bound dPPR proteins

To elucidate the atomic mechanism of ssRNA recognition by dPPR proteins, we launched a systematic effort to obtain the crystal structure of functional dPPR in complex with target RNA. On the basis of the hypothesis that the number of dPPR repeats might influence crystallization by affecting molecular packing, we altered the number of dPPR repeats and purified many batches of dPPR proteins with different repeat numbers for crystallization. After numerous unsuccessful trials, we finally succeeded in crystallizing each dPPR-U_8_N_2_ in complex with its respective target ssRNA (5′-UUUUNNUUUU-3′), in space group P2_1_2_1_2_1_ (Methods). These four structures were determined by molecular replacement (MR) using the atomic coordinates of the consensus PPR^29^ (cPPR, PDB accession code 4PJR), and were refined to resolutions of 2.20, 2.30, 2.60 and 2.53 Å, for dPPR-U_10_, dPPR-U_8_C_2_, dPPR-U_8_A_2_ and dPPR-U_8_G_2,_ respectively (Table 1). When dPPR-U_8_C_2_ was superimposed on dPPR-U_10_, dPPR-U_8_A_2_ and dPPR-U_8_G_2_, the root mean square deviations were 0.73, 0.99 and 1.00 Å over 348, 367 and 374 Cα atoms, respectively (Supplementary Fig. 4b). Because the four structures exhibited almost identical features except for the RNA base binding details of repeats 5 and 6, we focused on describing the structure of RNA-bound dPPR-U_8_C_2_.

### RNA-bound dPPR adopts a right-handed superhelical structure

**Figure 2:**
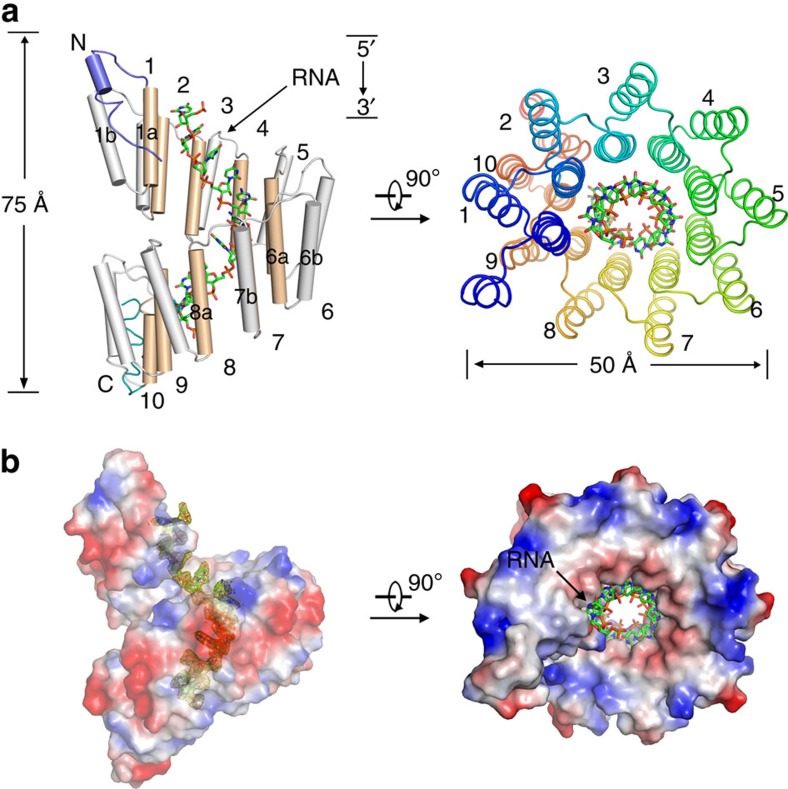
Overall structure of RNA-bound dPPR-U_8_C_2_. (**a**) Overall structure of dPPR-U_8_C_2_ bound to its target RNA element. dPPR-U_8_C_2_ comprises 10 repeats capped by a small NTD helix (slate) and a CTD helix (cyan). The 10 dPPR repeats of dPPR-U_8_C_2_ form a right-handed superhelical assembly. Wheat and grey bundles indicate helix a and helix b, respectively. (**b**) Electron density of target RNA is clearly visible in the cavity of the dPPR superhelix. The electron density, contoured at 1σ, is shown in yellow. The surface electrostatic potential was calculated with PyMOL. Two perpendicular views are presented, with the ssRNA molecule depicted as sticks. All structure figures were prepared using PyMOL.

In the complex structure, dPPR-U_8_C_2_ has 10 dPPR repeats (residues 174–523), which are capped by NTD and CTD helices (Fig. 2a). Each repeat in dPPR-U_8_C_2_ contains 35 amino acids, forming a hairpin of α-helices that both contain four helical turns followed by a five-residue loop. The two helices, formed by residues 1–14 and 17–30 (Fig. 1a), are designated as helix a and helix b, respectively (Fig. 2a, left panel). The whole-protein molecule has an overall appearance of a solenoid with a polar axis of 75 Å and a diameter of  50 Å (Fig. 2a). The internal layer along the superhelical axis is constituted by helices a, whereas helices b outline the external layer of the superhelix. Following the assignment of dPPR proteins in the electron density maps, electron densities indicative of RNA bases, which interdigitated with PPR helices, emerged in the cavity of the superhelix (Fig. 2b and Supplementary Fig. 4c). Because of the limited quality of the electron density data, only the 10 nucleotides coordinated by repeats could be modelled. Only one complex comprising one dPPR molecule with an ssRNA target was present in each asymmetric unit, similarly to the solution complex structure of *ATPH*-bound PPR10^30^. The overall dPPR protein structure consists of repetitions of helix pairs packing against each other to form a right-handed superhelical spiral shell that embraces its target ssRNA. The ssRNA molecule forms a right-handed parallel duplex structure with an ‘outer-layer' spiral protein enclosure. All 10 nucleotides in the target RNA elements strictly exhibit the modular pattern binding to corresponding dPPR repeats.

### Structural explanation of conformational plasticity of dPPR

**Figure 3:**
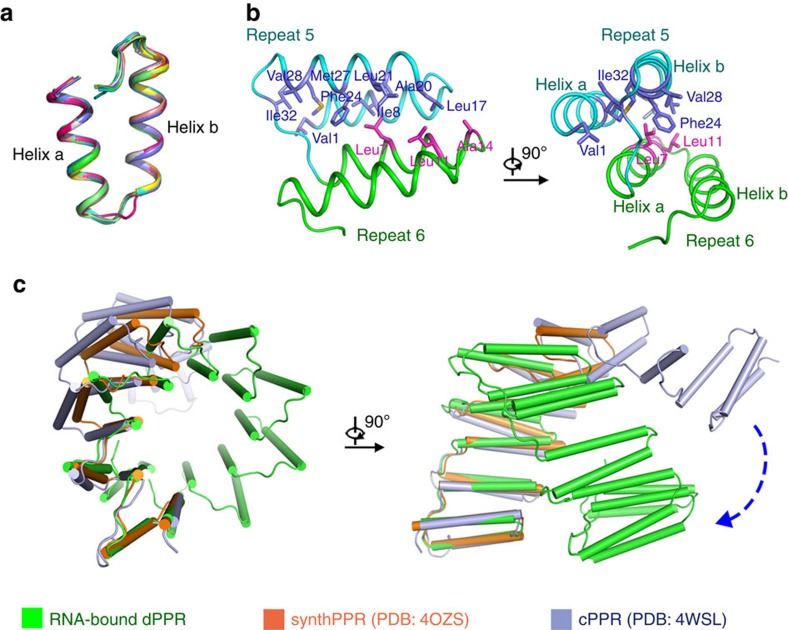
Structural plasticity of U_8_C_2_. (**a**) All dPPR repeats of dPPR-U_8_C_2_ exhibit a nearly identical conformation. Each repeat is organized into two helices (a and b) followed by a short loop. (**b**) The contact between adjacent dPPR repeats is primarily mediated by van der Waals interactions. Repeats 5 and 6 from dPPR-U_8_C_2_ are coloured cyan and green, respectively. Two perpendicular views are presented, with amino acids that participate in the interaction shown in violet and magenta at repeats 5 and 6, respectively. (**c**) RNA-bound dPPR-U_8_C_2_ exhibits a more compressed conformation than RNA-free cPPR and *synth*PPR3.5. The three structures are superimposed using the first PPR repeat of each protein, and dPPR-U_8_C_2_, cPPR and *synth*PPR3.5 are coloured green, light blue and orange. The blue dashed arrow indicates the conformational differences between dPPR-U_8_C_2_ and cPPR.

Similarly to RNA-free PPR10, all dPPR-U_8_C_2_ repeats exhibit a nearly identical conformation except for the short turns connecting helix a and helix b (Fig. 3a). In addition, all dPPR repeats exhibit a high degree of structural homology with the repeats in RNA-bound PPR10 (PDB ID: 4M59)^22^, artificially engineered cPPR-polyC (PDB ID: 4WSL)^29^ and synthetic PPR protein *synth*PPR3.5 (PDB ID: 4OZS)^35^, indicating that our RNA-bound dPPR motifs fold similarly to the natural ones and to engineered proteins in their RNA-free form. In the dPPR-U_8_C_2_ structure, each helix b stacks against the helix a of the following repeat through extensive van der Waals interactions, forming an inter-repeat structure similar to a three-helix bundle (Fig. 3b). Furthermore, although the structures of cPPR-polyC, *synth*PPR3.5, RNA-bound PPR10 (repeat 6–15) and dPPR-U_8_C_2_ all exhibit superhelical spiral shapes, they are distinct from one another in their configurational details. Although it has a similar diameter to RNA-free cPPR-polyC and *synth*PPR3.5, RNA-bound dPPR-U_8_C_2_ exhibits a more compact conformation with a helical period length of ∼70 Å, which is shorter than that of cPPR proteins (∼90 Å; Fig. 3c). The fact that the three types of engineered PPR motifs (dPPR, cPPR and *synth*PPR) are almost identical indicates that RNA binding may change the interaction between PPR hairpin-shaped motifs and induce conformational changes. Compared with RNA-bound PPR10 (repeats 6–15), RNA-bound dPPR-U_8_C_2_ also exhibits in a tighter form, and there is a 20-Å difference between their diameters (Supplementary Fig. 5). According to previous studies, repeats 6–15 of PPR10 fail to bind RNA perfectly, thus suggesting that RNA binding might contribute to subtle conformational variations. We speculate that these differences may be gradually amplified over an increasing number of repeats, ultimately leading to the prominent compression of the superhelix. This conformational plasticity appears to be a result of extensive van der Waals interactions between adjacent repeats. A similar phenomenon has been observed in DNA-bound/unbound TALE crystal structures^36^,^37^ but not in the PUF–RNA interaction^8^,^38^.

### Structural basis for specific RNA recognition by PPR code

**Figure 4:**
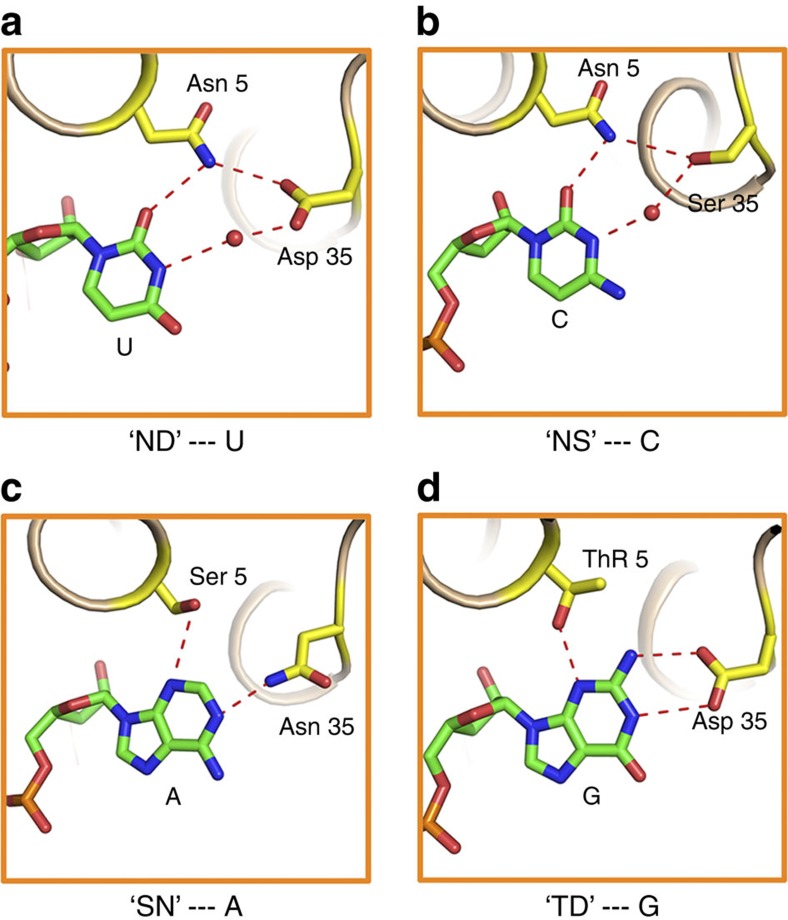
Structural basis of nucleobase recognition by dPPR repeats. PPR code amino acids selectively target nucleotides by forming direct or indirect hydrogen bonds to the Watson–Crick faces of bases. The specific recognition patterns of the bases U (**a**), C (**b**), A (**c**) and G (**d**) by dPPR repeats are shown in the zoom-in view. The side chains of the 5th and 35th residues in each PPR repeat are shown in yellow. Bases are labelled and coloured according to atom type (carbon: green, oxygen: red, nitrogen: blue). The hydrogen bonds are represented by red dotted lines. Water molecules are represented by red spheres. Single-letter abbreviations for the amino acid residues and nucleobases are as follows: D, Asp; N, Asn; S, Ser; T, Thr; A, Adenine; C, Cytosine; G, Guanine and U, Uracil.

As observed in all four complex structures, four types of dPPR repeats recognize their corresponding targets by forming hydrogen bonds with the Watson–Crick faces of the nucleotides, which explains why PPR proteins bind ssRNAs instead of double stranded ones. The electron densities of the RNA nucleotides 5 and 6 differ remarkably from each other (Supplementary Fig. 6), providing insight into the base-recognition mechanisms of PPR repeats. Previous studies have strongly suggested that the polar amino acid at the 5th position in each repeat is the chief determinant of RNA base specificity. Serine or threonine at this position results in a preference for purines, whereas the presence of asparagine is correlated with a preference for pyrimidines. The structures of RNA-bound dPPRs provide a powerful explanation for these codes. The amide group of the Asn5 side chain donates a hydrogen bond to the O2 atom of the corresponding pyrimidine, whereas the N3 atom of purine accepts a hydrogen bond from the hydroxyl group of the corresponding amino acid (Fig. 4). The 35th residue, which is the second significant amino acid of the PPR code, is also located in close proximity to the corresponding nucleobase. We observed that water molecules between bases and PPR repeats mediate hydrogen bonds between the polar residues and the bases in the cases of uracil and cytosine recognition. This recognition pattern has not been reported in the TALE–DNA^36^,^37^ or PUF–RNA interactions^8^,^38^. Each water molecule between the base and corresponding PPR repeat forms two hydrogen bonds: one with the N3 atom of the pyrimidine and one with the carboxyl group of Asp35 (Fig. 4a) or the hydroxyl group of Ser35 (Fig. 4b). Base selectivity is determined via ‘water bridge' polarity. The N3 atom of uracil is a hydrogen bond donor, whereas the N3 atom of cytosine is a hydrogen bond acceptor. For purine, Asn35 or Asp35 form one (Fig. 4c) or two (Fig. 4d) hydrogen bonds with adenine and guanine, respectively. The N1 atom of adenine is a hydrogen bond acceptor, whereas both the N1 and N2 atoms of guanine are hydrogen bond donors. These structures demonstrate how the amino acids asparagine and aspartate at the 35th position contribute to purine base selectivity.

### Delineation of new PPR codes

**Figure 5:**
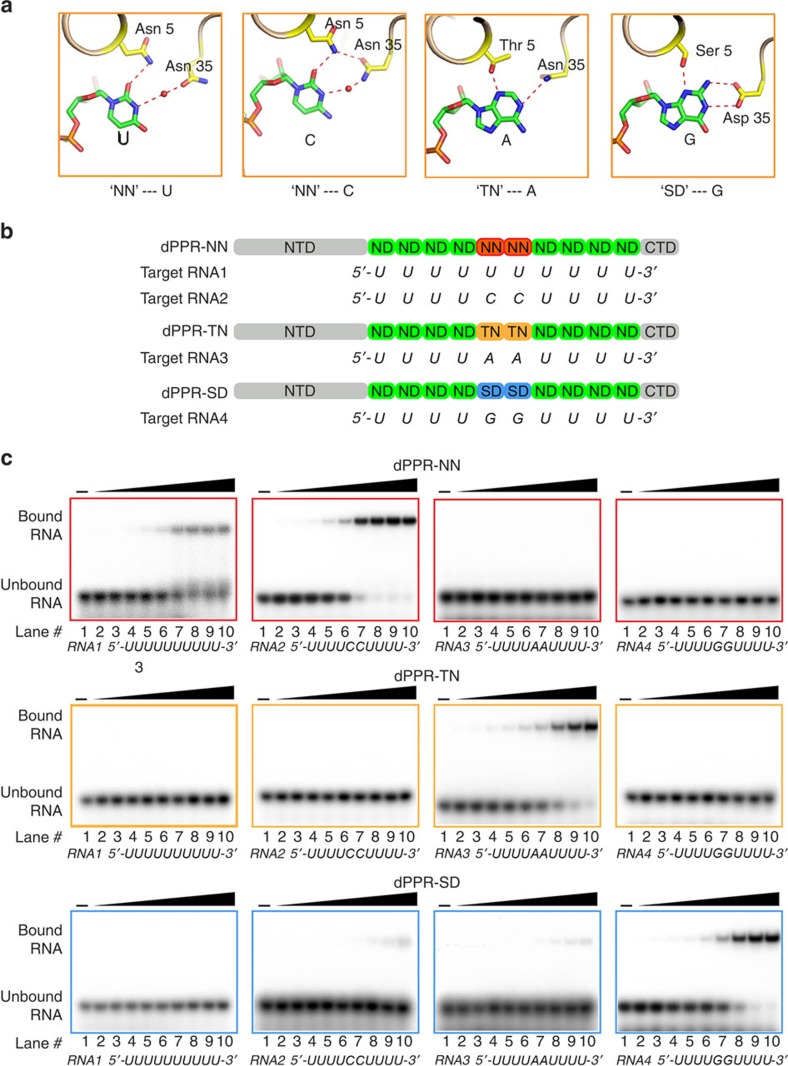
Interaction models for more PPR codes. (**a**) Prediction of another 4 interaction models for PPR codes ‘NN', ‘TN' and ‘SD' with U, C, A and G, respectively. The hydrogen bonds are represented by red dotted lines. Water molecules are represented by red spheres. (**b**) Schematic representation of dPPR-NN, dPPR-TN, dPPR-SD and their target RNA sequences. The shaded binary amino acids are indicative of PPR repeats with different codes. NTD and CTD are from native PPR10. (**c**) EMSA demonstrates the specific RNA recognition of dPPR proteins with predicted codes. The final concentrations of dPPR in lanes 1–10 are 0, 0.8, 1.6, 3.2, 6.25, 12.5, 25, 50, 100 and 200 nM, respectively. The detailed *K*_d_ values are shown in Supplementary Table 1.

In nature, the PPR code is degenerate^15^,^25^,^26^,^27^. Multiple combinations of amino acids at positions 5 and 35 can specify the same nucleotide, but sometimes the same combination of amino acids is similarly compatible with more than one type of nucleotide. Although we have not yet obtained crystal structures of PPR repeats with all code combinations, base-recognition models for codes such as NN, TN and SD^26^,^27^ can be rationally deduced from the existing structural information (Fig. 5a). For instance, the code NN has been reported to be equally compatible with uracil and cytosine. Water-mediated hydrogen bonds may connect the amino acid at the 35th position with its coordinating pyrimidine. The ability of the side chain of Asn35 to rotate is also important because it allows the amino acid to be a hydrogen bond donor or acceptor. Under these circumstances, we predict that the N3 atom of either uracil or cytosine may form a hydrogen bond with the water molecule but with the opposite polarity. We designed and purified proteins containing predicted codes NN, TN and SD for biochemical verification (Fig. 5b). The biochemical results corroborated the models, demonstrating that the code NN exhibits similar selectivity towards U and C with dissociation constants of ∼15 nM, whereas codes TN and SD specifically recognize A and G, respectively (Fig. 5c). Thus, these results provide a framework for deciphering the RNA targets of the mysterious PPR-motif code, which comprises more than 30 code combinations in nature^15^,^26^,^27^.

## Discussion

As a larger family of sequence-specific RNA-binding proteins, PPRs play various important roles in all aspects of organelles' RNA metabolism^15^. The dimerization states of PPR may function in the recognition of multiple RNA targets and the regulation of different signal responses. For example, PPR4 and PPR5 exist as monomers^39^,^40^, whereas HCF152 and PPR10 has been identified as homodimers^21^,^22^,^26^,^41^. Recently, two crystal structures of PPR in complex with RNA (*PSAJ*-bound PPR10 and *YCF3*-bound THA8) have revealed atypical modular and dimeric RNA-targeting modes^22^,^24^, whereas the solution structure of *ATPH*-PPR10 is monomeric^30^. In this study, each RNA–dPPR complex exhibited a monomeric and ideally modular RNA-binding mode, suggesting that the monomeric RNA-binding form of PPR is highly favourable under physiological conditions^15^.

Our data reveal that a dPPR protein recognizes its specific ssRNA target via hydrogen-bond-mediated interaction between the dPPR repeats and their coordinating nucleobases (Fig. 4). The most important feature reflected by our structures is that the amino acids at positions 5 and 35 in each repeat, which interact directly or indirectly with the target nucleobase, are critical for base selection. This finding provides structural evidence for PPR code theory, corroborating previous biochemical and computational results^25^,^26^,^27^. In addition, on the basis of our structures, the specific base recognition of PPR codes such as NN, TN and SD can be explained, thus providing a reference for bi-residue combinations. Given the dimension discrepancy between purine and pyrimidine, a PPR repeat that contains small amino acids such as alanine or glycine at position 5 can accommodate purine without notable steric clash but exhibits little specificity, potentially resulting in weak RNA-binding activity compared with ‘TD' and ‘SN'. Previous functional studies have focused on only a few PPR members because of the limitation imposed by RNA target identification. Herein, deciphering the codes for RNA recognition through use of dPPRs enabled the precise prediction of the RNA targets of numerous uncharacterized PPR proteins, and may provide a comprehensive understanding of the PPR family^15^,^27^.

Furthermore, our high-resolution structures provide precise information about RNA coordination by PPR repeats. Several additional important amino acids in dPPR repeats, such as Val2 and Lys13 (Supplementary Figs 7 and 8), also contribute to RNA binding. Together with its counterpart in the next repeat, each Val2 clamps its corresponding nucleobase in a sandwich-like manner through van der Waals interactions (Supplementary Fig. 7b), thus, explaining why the amino acids at this position are usually hydrophobic, as reported in a previous study^22^. Another crucial amino acid involved in RNA binding is the lysine at position 13. Each phosphate group of the target ssRNA is oriented to helix a. Lys13 is positioned at the extremity of helix a in each repeat, contributing to the positive electrostatic potential facilitating interactions with the negatively charged phosphate (Supplementary Fig. 8a). Interactions with the phosphate group of ssRNA, which are invariant for repeats 1 through 8, are mediated by salt bridges (Supplementary Fig. 8b) between Lys13 of repeat 1 and the 5′ phosphate group of U3, and between Lys13 of repeat 8 and the 5′ phosphate group of U10. Notably, Lys13 of repeat 9 also forms a salt bridge with the 3′ phosphate group of U10, but no interaction was observed between Lys13 of repeat 10 and ssRNA because of the poor electron density of the 3′-terminal RNA nucleotides. The substitution of Lys13 with alanine in each repeat completely abolished RNA binding (Supplementary Fig. 8c), consistently with the results from a previous report^29^. These detailed analyses emphasize that the 33 other residues in addition to the two code residues must be considered when optimizing designed PPR repeats in future work.

Together, our structural analyses should help to improve the mechanistic perception of the PPR protein and facilitate the optimal design of useful tools for RNA manipulation with enhanced specificity and affinity. Furthermore, our work may serve as a model to explore the α-helical repeat protein universe *in silico*^42^,^43^. Moreover, the detailed elucidation of the interaction mechanism between dPPR repeats and different nucleobases will allow the development of new types of dPPRs targeting modified nucleobases, including N6-methyladenosine^44^ and pseudouridine^45^,^46^,^47^, for potential biotechnological applications.

## Methods

### Protein preparation

All customized PPR genes were synthesized by Genewiz (GENEWIZ, Inc., China) and then subcloned into the pET21b vector (Novagen), resulting in recombinant dPPR proteins fused with a 6 × His tag at the C-termin. The plasmids were transformed into *E. coli* BL21(DE3). One litre lysogeny broth medium supplemented with 100 μg ml^−1^ ampicillin was inoculated with a transformed bacterial pre-culture and shaken at 37 °C until the optical density at 600 nm reached 1. The culture was cooled to 16 °C and induced with 0.2 mM isopropyl-β-D-thiogalactoside. After growing for 16 h at 16 °C, the bacterial pellet was collected and homogenized in buffer A (25 mM Tris–HCl, pH 8.0, 150 mM NaCl). After sonication and centrifugation at 23,000*g* at 4 °C, the supernatant was loaded onto a column equipped with Ni^2+^affinity resin (Ni-NTA, Qiagen), washed with buffer B (25 mM Tris–HCl, pH 8.0, 150 mM NaCl, 15 mM imidazole), and eluted with buffer C (25 mM Tris–HCl, pH 8.0, 250 mM imidazole) followed by ion exchange (Source 15Q, GE Healthcare). Each protein was then subjected to gel filtration chromatography (Superdex-200 10/300, GE Healthcare). The buffer for gel filtration contained 25 mM Tris–HCl, pH 8.0, 100 mM NaCl, 5 mM MgCl_2_ and 5 mM 1,4-dithiothreitolDTT (Supplementary Fig. 9). The peak fractions were incubated with target RNA oligonucleotides at a molar ratio of ∼1:1.5 at 4 °C for ∼40 min before crystallization trials.

### Crystallization

To obtain crystals of dPPR–RNA complexes, we first examined various combinations of dPPR-U_10_ boundaries and corresponding RNA oligonucleotides (Takara). Finally, dPPR-U_10_ (residues 123–572) and 18-nt RNA 5′-ggggUUUUUUUUUUcccc-3′ were crystallized in the reservoir solution 11–13% (w/v) polyethylene glycol 3,350, 100 mM Bis-Tris propane, pH 6.5, 150 mM MgCl_2_. However, the crystals exhibited poor diffraction, diffracting only to 8 Å. To generate dPPR-U_10_ crystals with good X-ray diffraction, two rounds of additive screening were performed. The first additive screen revealed that ethyl acetate improved the diffraction to 3.5–4 Å. In this context, a second additive screen was then performed. Finally, the best crystals were obtained under the following conditions: 11–13% (w/v) polyethylene glycol 3,350, Bis-Tris propane, pH 6.5, 150 mM MgCl_2_, 1.5% ethyl acetate and 3% (w/v) D-(+)-glucose monohydrate. The crystals were flash frozen in liquid nitrogen using a 2 × mother solution as the cryoprotective buffer and diffracted beyond 2.3 Å at Shanghai Synchrotron Radiation Facility (SSRF) beamline BL19U.

The other three proteins (dPPR-U_8_C_2_, dPPR-U_8_A_2_ and dPPR-U_8_G_2_; residues 123–572) and their corresponding 18-ntRNAs with sequences of

5′ ggggUUUUCCUUUUcccc 3′

5′ ggggUUUUAAUUUUcccc 3′

5′ ggggUUUUGGUUUUcccc 3′

yielded crystals in the same reservoir solution. All RNA-bound dPPR proteins were crystallized by the hanging-drop vapour-diffusion method at 18 °C and mixed 1 μl sample with an equal volume of reservoir solution. Crystals appeared overnight and grew to full size within 5–9 days.

### Data collection and structural determination

All data sets were collected at SSRF beamline BL19U or BL17U and processed with the HKL3000 or HKL2000 packages^48^. Further processing was performed with programs from the CCP4 suite^49^. Data collection and structure refinement statistics are summarized in Table 1. The structure of the dPPR–RNA complex was solved by MR with the newly solved RNA-free structure as the search model using the programme PHASER^50^. The structure was manually iteratively refined with PHENIX and COOT^51^,^52^ (Table 1).

### Electrophoretic mobility shift assay (EMSA)

The ssRNA oligonucleotides were radiolabelled at their 5′ ends with [γ-^32^P] ATP (PerkinElmer), catalysed by T4 polynucleotide kinase (Takara). For EMSA, dPPR proteins were incubated with ∼2 nM ^32^P-labelled probe in final binding reactions containing 25 mM Tris–HCl, pH 8.0, 5 mM MgCl_2_, 5 mM 1,4-dithiothreitol, 0.1 mg ml^−1^ bovine serum albumin, 50 ng ml^−1^ heparin and 10% glycerol for 20 min on ice. The reactions were then resolved on 8% native acrylamide gels (37.5: 1 acrylamide: bis-acrylamide) in 0.5 × Tris-glycine buffer under an electric field of 15 V cm^−1^ for 40 min. Gels were visualized on a phosphor screen (Amersham Biosciences) using a Typhoon Trio Imager (Amersham Biosciences). All presented images are representative of results from at least three independent experiments.

## Additional information

**Accession codes:** The atomic coordinates and structure factors for the four structures in complex with RNA have been deposited in the Protein Data Bank (PDB) with the accession codes 5I9F (poly-U), 5I9G (poly-UC), 5I9D (poly-UA) and 5I9H (poly-UG).

**How to cite this article:** Shen, C. *et al.* Structural basis for specific single-stranded RNA recognition by designer pentatricopeptide repeat proteins. *Nat. Commun.* 7:11285 doi: 10.1038/ncomms11285 (2016).

## Supplementary Material

Supplementary Information Supplementary Figures 1-9, Supplementary Table 1 and Supplementary References.

## Figures and Tables

**Table 1:** Statistics of data collection and refinement.

	**dPPR-U**_**10**_	**dPPR-U**_**8**_**C**_**2**_	**dPPR-U**_**8**_**A**_**2**_	**dPPR-U**_**8**_**G**_**2**_
*Data collection*				
Space group	P2_1_2_1_2_1_	P2_1_2_1_2_1_	P2_1_2_1_2_1_	P2_1_2_1_2_1_
Cell dimensions				
*a*, *b*, *c* (Å)	52.73, 85.27, 95.10	51.43, 84.78, 94.36	52.55, 84.90, 95.90	52.06, 85.10, 95.40
*α*, *β*, *γ* (°)	90.00, 90.00, 90.00	90.00, 90.00, 90.00	90.00, 90.00, 90.00	90.00, 90.00, 90.00
Resolution (Å)	40–2.20 (2.28–2.20)	40–2.30 (2.38–2.30)	47.7–2.60 (2.71–2.60)	47.7–2.50 (2.61–2.50)
*R*_merge_ (%)	6.8 (56.8)	7.2 (20.8)	10.0 (46.5)	8.2 (5.4)
*I*/*σ*	21.1 (2.6)	26.3 (3.2)	16.7 (5.5)	19.8 (4.9)
Completeness (%)	99.0 (99.0)	98.4 (87.9)	99.8 (98.3)	99.8 (98.5)
Redundancy	3.7 (3.4)	6.0 (5.8)	8.6 (10.9)	10.1 (12.8)
				
*Refinement*				
Resolution (Å)	38.90–2.19	38.67–2.29	46.08–2.60	47.70–2.50
No. reflections	22,260	18,753	13,769	15,113
*R*_work_/*R*_free_ (%)	25.03/29.94	22.51/24.75	26.69/29.30	22.30/29.60
No. atoms				
Protein	2,990	2,860	2,778	2,850
Ligand/ion	204	208	198	225
Water	76	67	41	49
B-factors				
Protein	56.4	53.3	52.0	57.1
Ligand/ion	44.2	49.3	41.3	49.5
Water	54.1	52.6	47.2	46.8
R.m.s. deviations				
Bond lengths (Å)	0.010	0.016	0.015	0.007
Bond angles (°)	1.241	1.802	1.793	0.917

R.m.s., root mean square.

Values in parentheses are for the highest resolution shell. *R*_merge_=Σ_*h*_Σ_*i*_|*I*_*h*,*i*_−*I*_*h*_|/Σ_*h*_Σ_*i*_*I*_*h*,*i*_, where *I*_*h*_ is the mean intensity of the *i* observations of symmetry related reflections of *h*. *R*=Σ|*F*_obs_−*F*_calc_|/Σ*F*_obs_, where *F*_calc_ is the calculated protein structure factor from the atomic model (*R*_free_ was calculated with 5% of the reflections selected).
