# Supplementary material for: Structural basis for specific single-stranded RNA recognition by designer pentatricopeptide repeat proteins
Source: Nat Commun. 2016 Apr 18;7:11285. doi: 10.1038/ncomms11285 (PMC4837458; doi:10.1038/ncomms11285)
Supplement: Supplementary Information — Supplementary Figures 1-9, Supplementary Table 1 and Supplementary References. [file ncomms11285-s1.pdf]

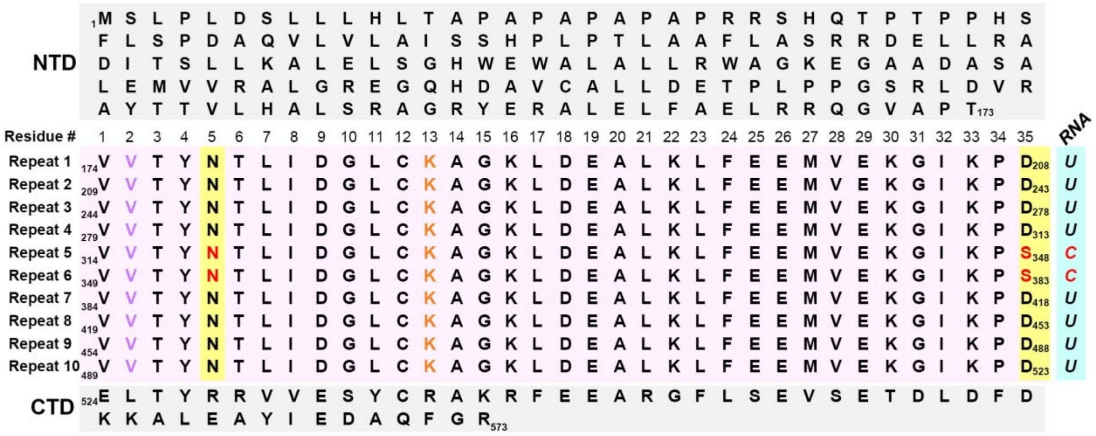

Supplementary Fig. 1 | Primary sequence of dPPR protein. The variable PPR code amino acids of repeats 5 and 6 are shaded in yellow and the corresponding nucleotides of RNA are coloured red. The other residues at positions 2 and 13 that also contribute to RNA binding are lilac and orange, respectively. The corresponding RNA sequence recognized by the repeats is listed on the right, 5′ to 3′ from top to bottom.

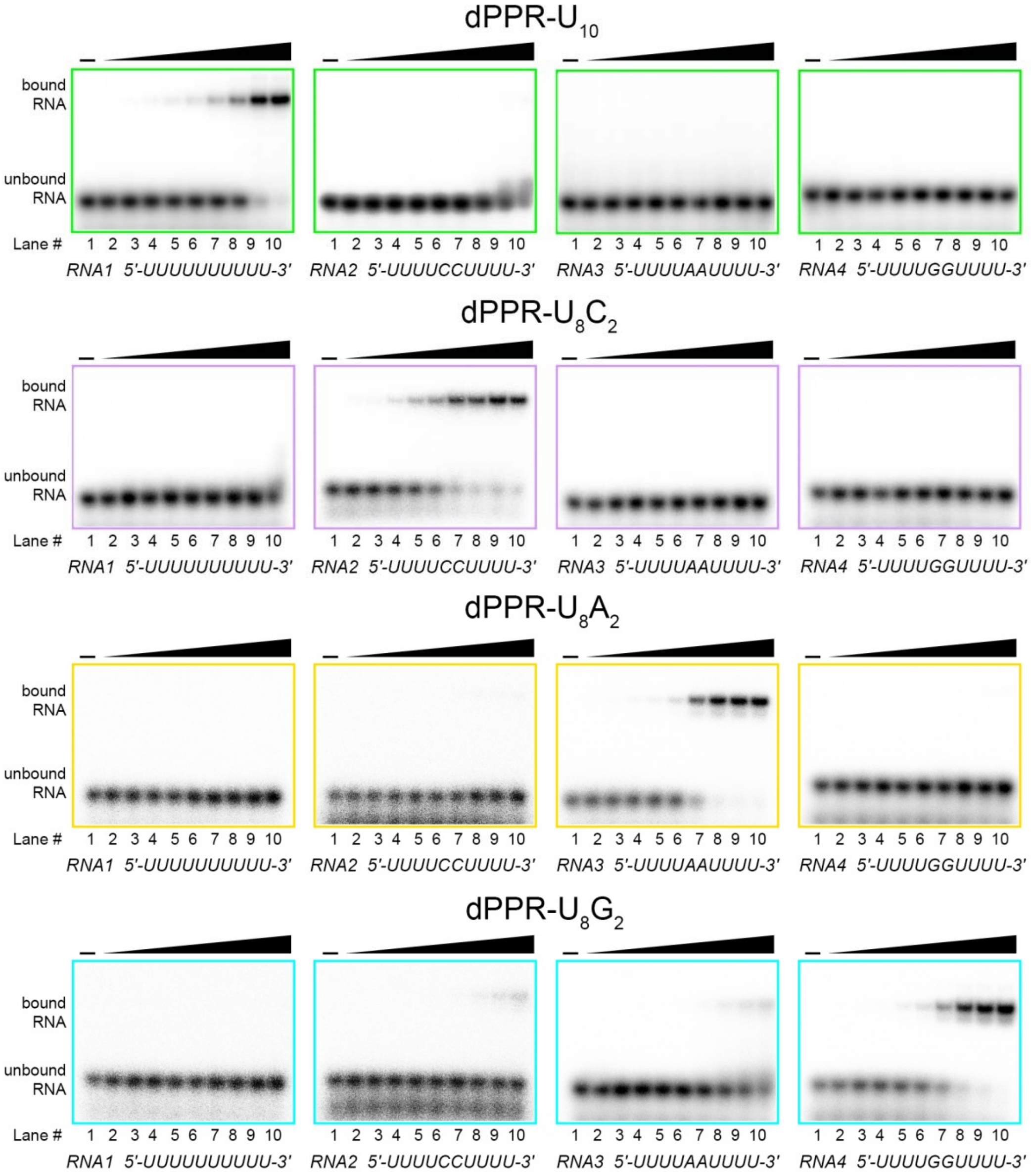

Supplementary Fig. 2 | Specific RNA target binding of dPPR-U_{10}, dPPR-U_8C_2, dPPR-U_8A_2, and dPPR-U_8G_2. EMSA demonstrates the specific binding of dPPR-U_{10}, dPPR-U_8C_2, dPPR-U_8A_2, and dPPR-U_8G_2 with variant RNA oligonucleotides. The final concentrations of dPPR in lanes 1-10 are 0, 0.8, 1.6, 3.2, 6.25, 12.5, 25, 50, 100, and 200 nM, respectively. The detailed K_d values are shown in Supplementary Table 1.

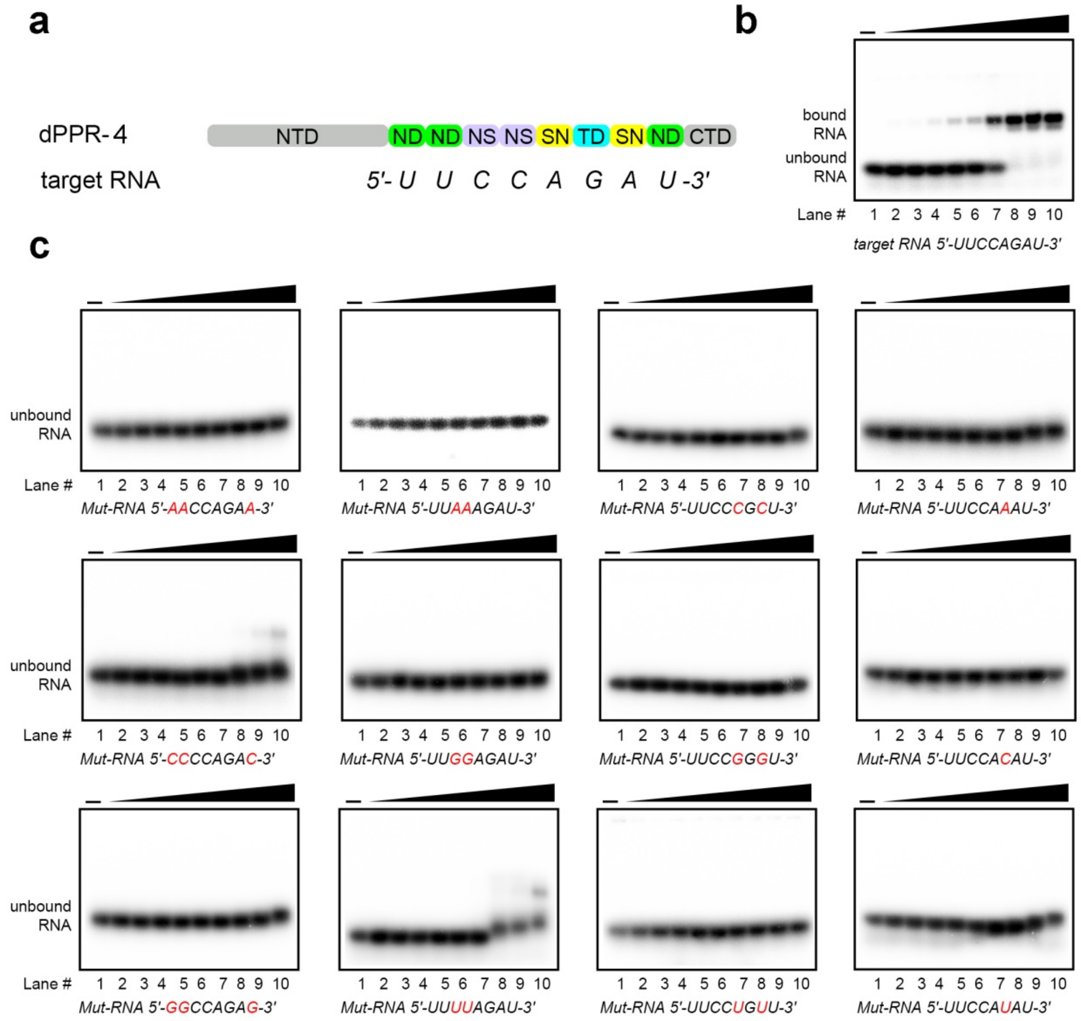

Supplementary Fig. 3 | Specific RNA target binding of dPPR-4. (**a**) Schematic view of dPPR-4 and its target RNA sequence. (**b**) and (**c**) EMSA demonstrates the specific binding of dPPR-4 with variant RNA oligonucleotides. The final concentrations of dPPR-4 in lanes 1-10 are 0, 0.8, 1.6, 3.2, 6.25, 12.5, 25, 50, 100, and 200 nM, respectively. Nucleotides that differ from the ones in the original target RNA are labelled in red. The detailed K_d values are shown in Supplementary Table 1.

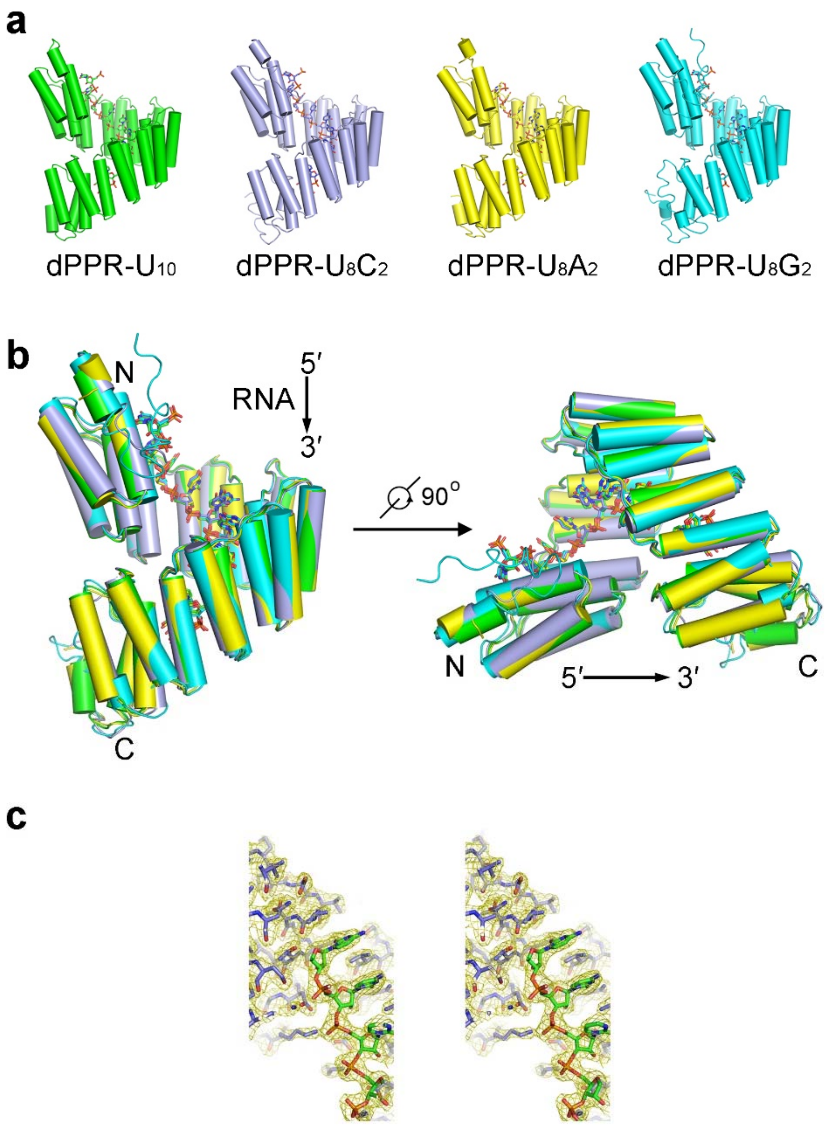

Supplementary Fig. 4 | Four RNA-bound dPPRs exhibit highly similar conformations. (**a**) Complexes dPPR-U_{10}, dPPR-U_8C_2, dPPR-U_8A_2, and dPPR-U_8G_2 are coloured green, light blue, gold and cyan, respectively. (**b**) Superposition of the four dPPR structures shows the small differences existing between our dPPR molecules: dPPR-U_8C_2 is superimposed on dPPR-U_{10}, dPPR-U_8A_2 and dPPR-U_8G_2, and the root mean square deviation (RMSD) values are 0.73 Å, 0.99 Å and 1.00 Å over 348, 367 and 374 Cα atoms, respectively. Two perpendicular views are presented, with the ssRNA molecule shown in sticks. All structure figures were prepared with PyMOL[1]. (**c**) Representative portion of the 2Fo-Fc electron density map for RNA-dPPR-U_8C_2, contoured at 1.0 σ and displayed in stereo.

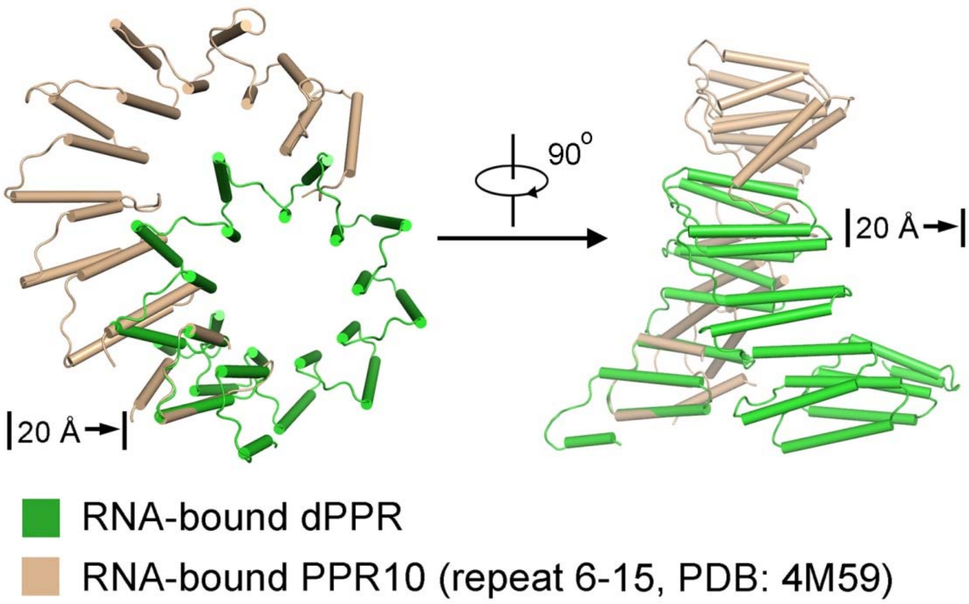

Supplementary Fig. 5 | RNA-bound dPPR and RNA-bound PPR10 (repeats 6-15) exhibit different conformations. Superposition of RNA-bound dPPR-U_8C_2 and RNA-bound PPR10 (repeats 6-15). The two structures are superimposed using the first repeat of dPPR-U_8C_2 and the 6th repeat of PPR10. RNA-bound PPR10 is shown for residues 280 to 630, comprising PPR repeats 6 to 15. RNA-bound dPPR-U_8C_2 and RNA-bound PPR10 (repeats 6-repeats 15)[2] are coloured green and wheat. Two perpendicular views are presented.

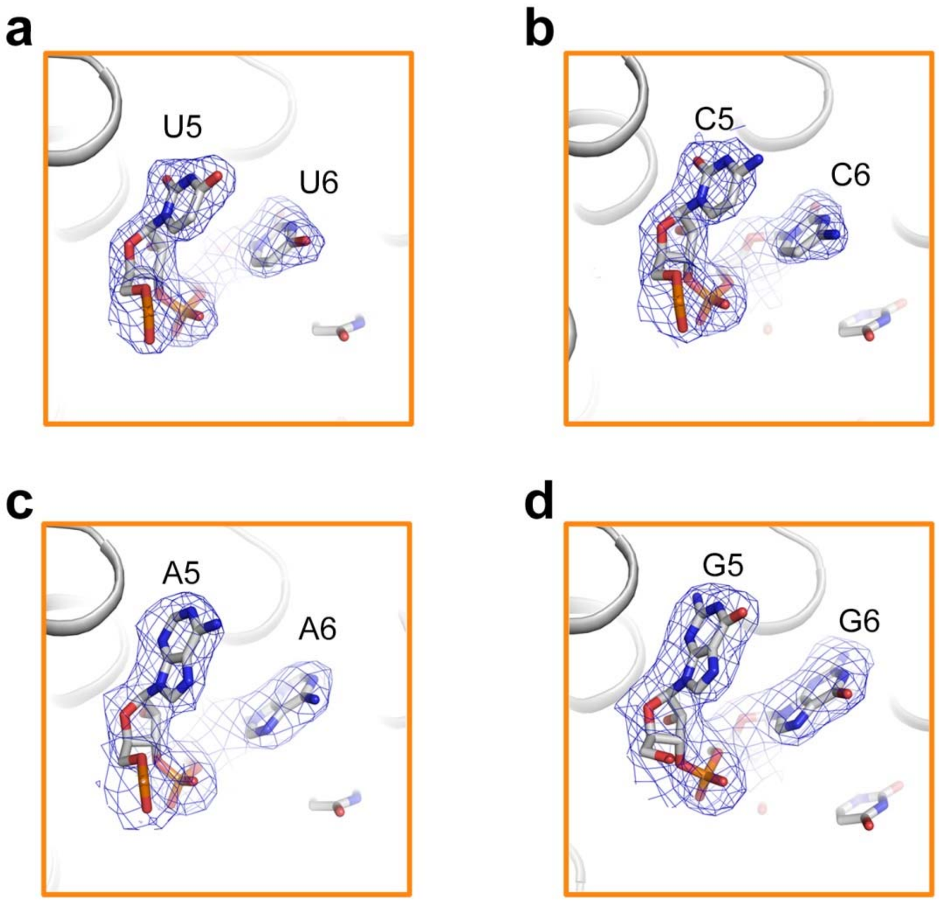

Supplementary Fig. 6 | Electron density maps for different RNA nucleotides. The 2Fo-Fc electron densities for nucleotides 5 and 6 of RNA U_{10} (**a**), U_8C_2 (**b**), U_8A_2 (**c**) and U_8G_2 (**d**). The electron densities, contoured at 1σ, are coloured blue.

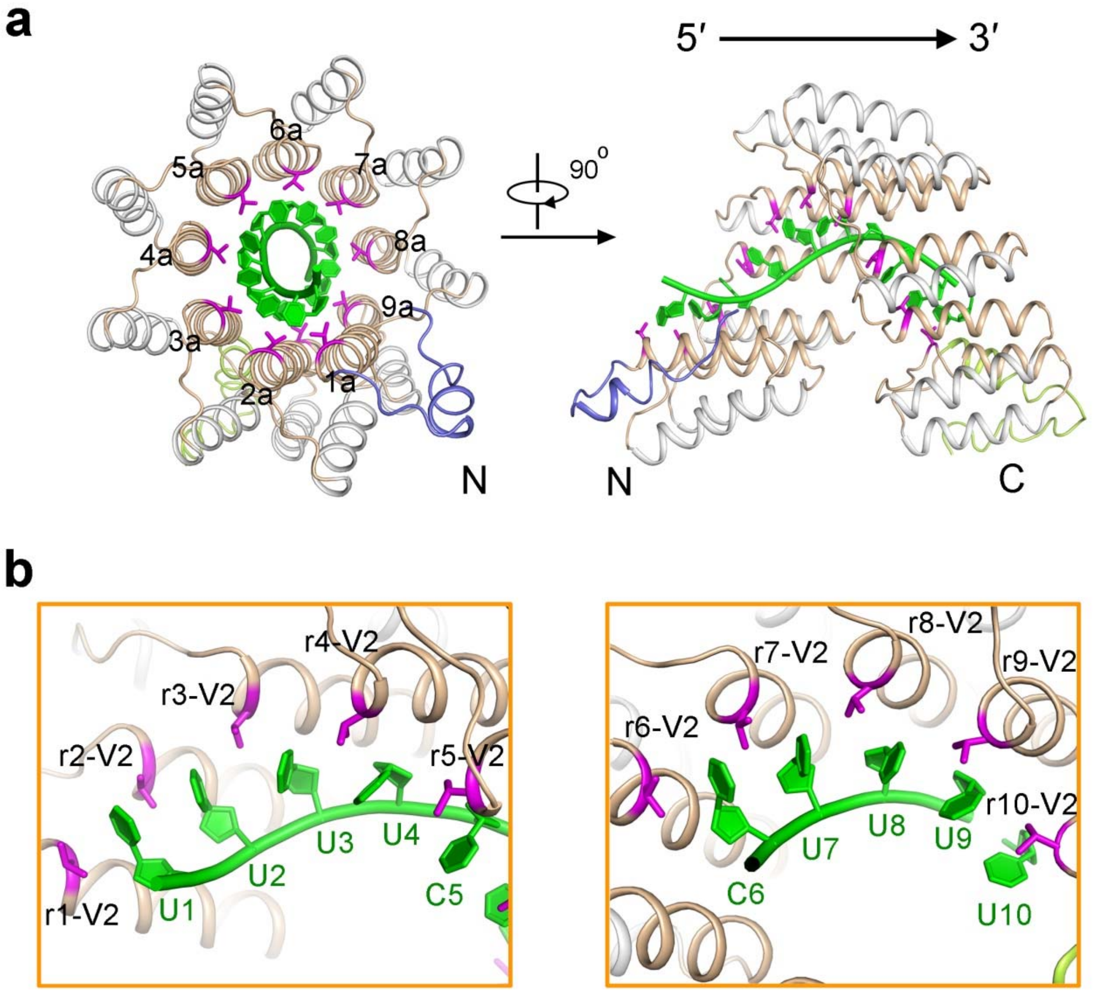

Supplementary Fig. 7 | Interaction patterns between Val2 in each repeat and its corresponding nucleobase. (**a**) Overview of interaction patterns in RNA-bound dPPR-U_8C_2. Two perpendicular views are presented, with the ssRNA molecule shown as a cartoon. (**b**) Zoom-in view of the Val2-base interaction. The RNA molecule is coloured green. Helix a and helix b are coloured wheat and grey, respectively. Val2 in each repeat is shown in magenta. The NTD helix and CTD helix are coloured purple and mint, respectively.

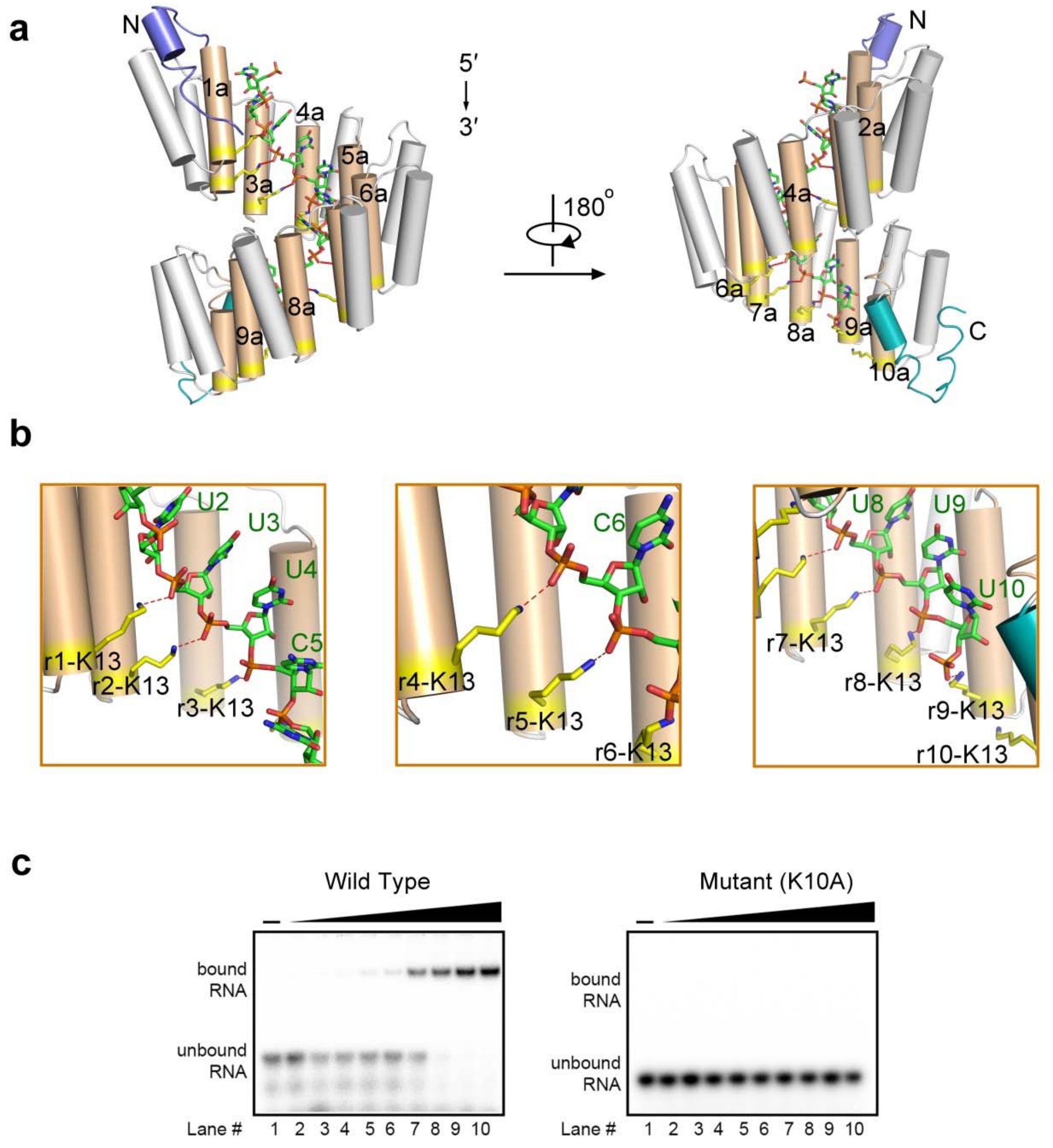

Supplementary Fig. 8 | Lys13 in each repeat binds to the backbone of the target RNA molecule. (**a**) Overview of the Lys13-RNA interaction in RNA-bound dPPR-U_8C_2. Wheat and grey bundles depict helices a and helices b, respectively. Lys13 (K13) in each repeat is coloured yellow. Two perpendicular views are presented, with the ssRNA molecule shown as sticks. (**b**) Zoom-in view of the Lys13-RNA interaction in RNA-bound dPPR-U_8C_2. The salt bridges are represented by red dotted lines. Nucleotides are labelled and coloured according to atom type (carbon: green, oxygen: red, nitrogen: blue, phosphate: orange). (**c**) Mutation of the dPPR protein (K10A, Lys-to-Ala substitution of each PPR repeat) abrogates the RNA binding ability. Protein concentrations were 0, 0.8, 1.6, 3.2, 6.25, 12.5, 25, 50, 100, and 200 nM in lanes 1-10, respectively.

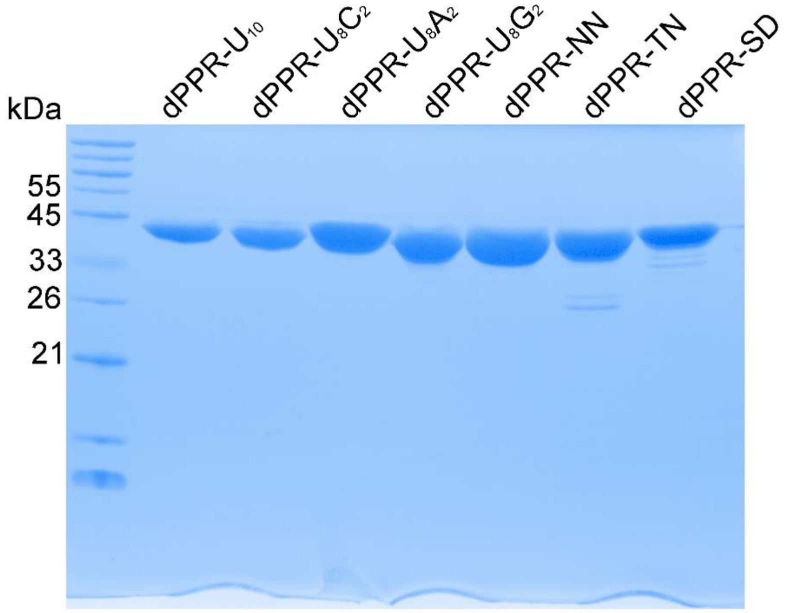

Supplementary Fig. 9 | The SDS-PAGE gel of purified dPPR proteins used in this study.

Designed PPRs	Target RNA sequences	Dissociation constants (nM)
dPPR-U_{10}	5′-UUUUUUUUUU-3′	74.3 ± 7.9
dPPR-U_8C_2	5′-UUUUCCUUUU-3′	18.5 ± 0.6
dPPR-U_8A_2	5′-UUUUAAUUUU-3′	23.8 ± 0.4
dPPR-U_8G_2	5′-UUUUGGUUUU-3′	29.8 ± 1.1
dPPR-NN	5′-UUUUUUUUUU-3′	15.9 ± 1.0
dPPR-NN	5′-UUUUCCUUUU-3′	14.6 ± 0.7
dPPR-TN	5′-UUUUAAUUUU-3′	58.9 ± 1.1
dPPR-SD	5′-UUUUGGUUUU-3′	31.1 ± 1.3
dPPR-4	5′-UUCCAGAU-3′	24.6 ± 1.6

Supplementary Table 1 | Apparent K_d values of dPPR-RNA complexes. The apparent K_d values were calculated using Quantity One V4.6 according to the results shown in Figure 5c, Supplementary Figure 2 and Supplementary Figure 3b.

Supplementary References

1. DeLano WL. The PyMOL molecular graphics system. (2002).
2. Yin P*, et al.* Structural basis for the modular recognition of single-stranded RNA by PPR proteins. *Nature* **504**, 168-171 (2013).
